# Light Management With Grating Structures in Optoelectronic Devices

**DOI:** 10.3389/fchem.2021.737679

**Published:** 2021-07-28

**Authors:** Wei Wang, Gong Wang, Yang Zhang, Xiang-Chao Sun, Yu Yu, Yudong Lian

**Affiliations:** ^1^ Center for Advanced Laser Technology, Hebei University of Technology, Tianjin, China; ^2^ Hebei Key Laboratory of Advanced Laser Technology and Equipment, Tianjin, China; ^3^ State Key Laboratory of Integrated Optoelectronics, College of Electronic Science and Engineering, Jilin University, Changchun, China; ^4^ Department of Experimental Pharmacology and Toxicology, School of Pharmacy, Jilin University, Changchun, China

**Keywords:** grating structures, optoelectronic devices, micro/nanostructure, nanoimprint lithography, light management

## Abstract

Ordered and patterned micro/nanostructure arrays have emerged as powerful platforms for optoelectronic devices due to their unique ordered-dependent optical properties. Among various structures, grating structure is widely applied because of its simple fabrication process, easy adjusting of size and morph, and efficient light trapping. Herein, we summarized recent developments of light management with grating structures in optoelectronic devices. Typical mechanisms about the grating structures in optoelectronic devices have been reviewed. Moreover, the applications of grating structures in various optoelectronic devices have been presented. Meanwhile, the remaining bottlenecks and perspectives for future development have been discussed.

## Introduction

There are many interesting and ordered micro/nanostructures in nature, organisms and plants, which play essential roles. The micro/nanostructures in lotus leaves can make water droplets roll freely (Zhang et al., 2012b); while rose petals can keep water droplets stay on it with the help of micro/nanostructures (Zhang et al., 2012a). The micro/nanostructures of reed leaves can guide the movement direction of water droplets (Wang et al., 2015). At the same time, more and more researches focus on applying micro/nanostructures in science. Ordered or patterned micro/nanostructure arrays have emerged as powerful platforms for cutting-edge applications due to their unique ordered-dependent properties, especially in optoelectronic devices, such as photodetectors, light emitting diodes, lasers, solar cells, bioelectronic, etc. The reasons why the micro/nanostructure arrays can be widely applied in many fields are they can enhance light scattering and reduce light reflection, improve the light extraction of organic light-emitting devices (OLEDs) and surface-to-volume ratio (Gao et al., 2021), produce photonic metasurfaces (Li et al., 2021). Various functions can be realized by adjusting the size, arrangement, and shape of each micro/nanostructure. Such as the light scattering effect can be improved by concave nanonets structure. Meanwhile, the antireflection effect can be realized by nanocone structure.

At present, the fabrication of excellent micro/nanostructure always depends on the development of nanofabrication technology, including templated method, lithographic technology, *in situ* preparation, direct laser writing, and self-assembly approaches. Many novel architectures have been fabricated through the above technologies to improve the performance of devices, such as microlens arrays, gratings, pyramid arrays, micro/nanorods, nanowire arrays, microsphere arrays, and so on (Zhmakin, 2011; Geng et al., 2014; Zhao and Ma, 2017). Microlens are arranged in 2D arrays to form an ordered array, micrometer or millimeter size usually are applied in light collimating (Song et al., 2013; Yu et al., 2013; Yuan et al., 2018). In addition, the microlens array can also enhance light trapping to improve the performance of optoelectronic devices (Choy et al., 2014; Kang et al., 2015). More importantly, they can be used in 3D imaging systems with large view angles, high temporal resolution, and so on (Yuan et al., 2018). Except for the microlens array, Chueh et al. reported the pyramid-patterned sapphire substrate could enhance the strong light interaction between MoS_2_ bilayers and the substrate to improve photodetector performance (Wang et al., 2017). Moreover, it has been speculated that a perovskite whispering gallery mode (WGM) microsphere array would have higher optical absorption for solar cells (Grandidier et al., 2011; Mihi et al., 2013). And with the help of 3D structures of nanowire and nanorod arrays, which make incident light undergo multiple scattering inside the structure. They are usually used in various solar conversion devices to enhance light-harvesting ability (Cho et al., 2011). Nanorod array also can be applied in the field of light-emitting diodes (LED), Chang et al. reported a kind of LED that could adjust the polarization of the emitted light with the help of nanorod arrays (Chou et al., 2018). Every structure has unique properties, while the gratings architectures are more popular due to the simple fabrication process, easy adjusting of size and morph, and excellent performance of light trapping. The grating structure has been applied in most optoelectronic devices and other cutting-edge applications. It’s necessary to summarize the researches about grating structure in optoelectronic devices.

In this minireview, we focus on the recent advancements in the application of grating structure in optoelectronic devices. Firstly, the typical light trapping mechanisms of grating structures in devices are discussed. And typical examples, such as the applications in photodetectors, solar cells, organic light-emitting devices, and lasers are summarized. Finally, the challenges and future perspectives for optoelectronic devices with grating structures are also discussed.

## Mechanism

**FIGURE 1:**
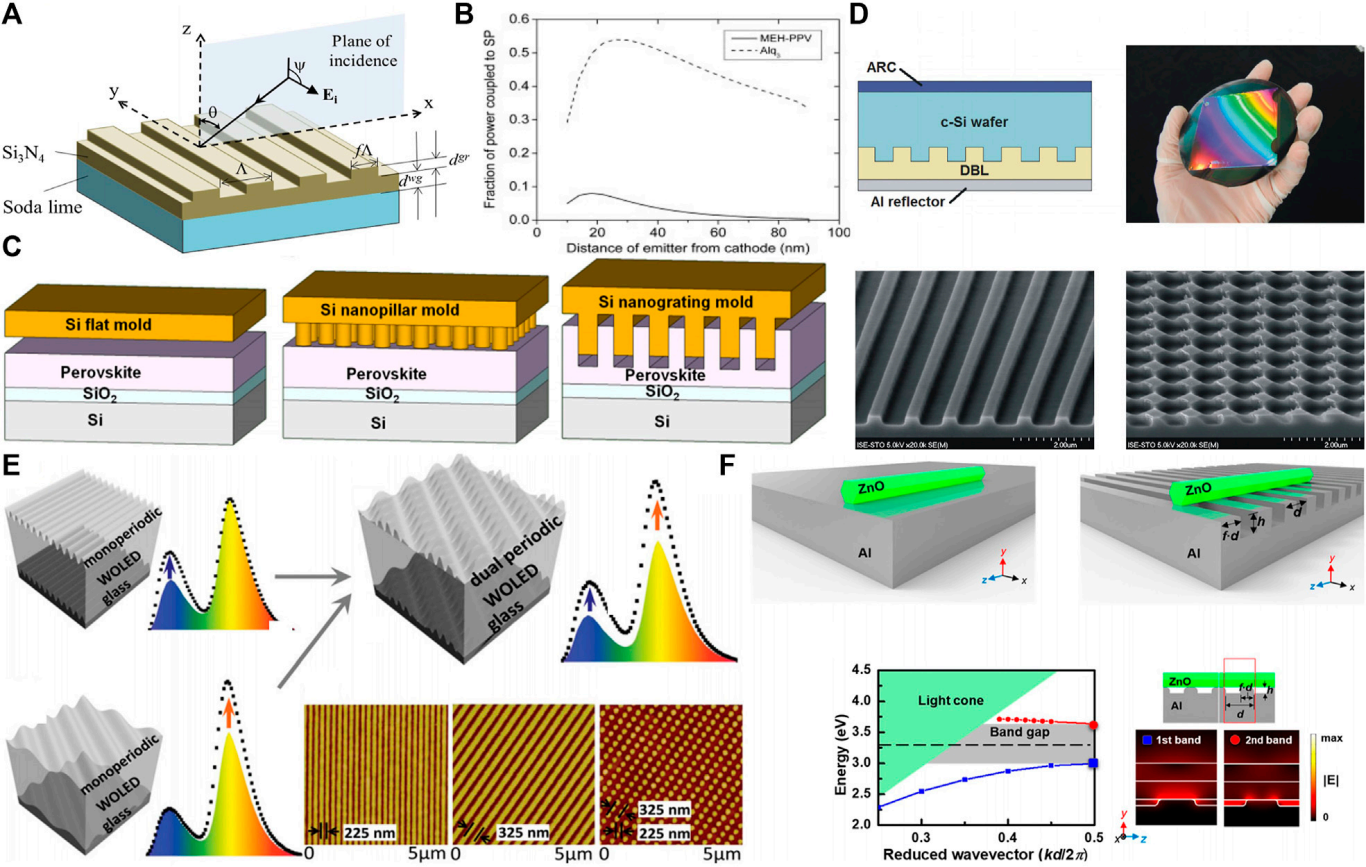
The grating structures in optoelectronic devices, **(A)** GMR filter with grating structure. **(B)** The calculated fraction of power lost from radiative excitons to surface plasmon modes is shown as a function of position of the exciton within the organic layer. **(C)**. Schematic of nanoimprint lithography process with different Si molds. **(D)** The schematic diagram, photograph and SEM of the grating structures in solar cell. **(E)** Schematic of the broadband light extraction using OLED with dual-periodic grating structures and the morphologies of a serious of grating structures. **(F)** Schematic representation and characteristics of surface plasmon waveguide and periodic grating structures. Reproduced from Cunningham et al. (2011) with permission of Optical Society of America. Reproduced from Barnes et al. (2002) with permission of WILEY. Reproduced from Hu et al. (2016) with permission of American Chemical Society. Reproduced from Mellor et al. (2013) with permission of Optical Society of America. Reproduced from Sun et al. (2013) with permission of WILEY. Reproduced from Lin et al. (2018) with permission of American Chemical Society.

Micro/nanostructures largely determined the performance of optoelectronic devices due to they can influence the optical properties of devices. However, it is important to balance the relationship between optical properties and electrical properties. The light management mechanisms of grating structure in optoelectronic devices are usually summarized as the resonant effect, plasmonic effect and scattering enhancements. Among various resonant strategies, the Mie theory is generally applied to spherical structures and whispering gallery mode (WGM) usually used to the sphere, toroid, and ring structures. These structures can limit light circulate at the periphery of the resonator. As for the grating structure, guided mode resonance (GMR) is applied. The normally incident plane wave can be coupled into a waveguide mode with the help of diffraction gratings (Yamada et al., 2017). The grating layer and supporting layer are necessary for GMR. They can induce sharp reflection and transmission anomalies. There will be a strong interaction between light and matter as long as it matches the outgoing emission or incoming excitation with the guided mode wavelengths (Collin, 2014). As shown in Figure 1A, Cunningham et al. fabricate Si_3_N_4_ periodic grating structures on a kind of soda-lime glass to form a GMR filter. While the GMR can be excited when the incident light satisfies Bragg diffraction by adjusting the period, depth of grating, and thickness of waveguide (Liu et al., 2011; Ko and Magnusson, 2018). Rational grating structures can induce plasmonic effects because these structures can redistribute optical fields and scatter light (Meinzer et al., 2014). Surface plasmon polariton (SPP) loss usually appeared in OLED devices, and the loss happened at the interface between dielectric and metal, which metal always serves as electrodes for OLED. And the metallic film can couple light to induce SPP modes, which transfer incident light into photo carriers (Atwater and Polman, 2010). Actually, SPP is a guided electromagnetic surface mode with transverse magnetic polarization. In general, the SPP modes coupled with excitons can’t support the energy couple out from the OLED devices for the traditional planar OLED with non-grating structures. As shown in Figure 1B, Barnes et al. have illustrated that the SPP loss was up to ∼40% for traditional planar OLEDs, resulting in the obvious limitation on the OLED development (Hobson et al., 2002). It is noticed that the grating structures within OLED devices can realize light extraction effectively. Such as the tunable grating array was fabricated on the metallic electrode to match the SPP mode with the energy and momentum of light along with the interface, leading to the occurrence of SPP resonance and increase of light extraction (Zayats et al., 2005). Besides the resonant effect and plasmonic effect, grating structures also can improve light scattering efficiently due to the structure prolong the optical path and increase the reflections and refractions (Han and Chen, 2010; Chi et al., 2017). The absorption ability always is limited by the Lambertian limit for films. However, light scattering will be increased among the micro/nanostructures when the size of each structure is larger than the wavelength. For example, the grating structures can produce multiple reflections and refractions of the incident light, leading to the prolongation of optical path length and the increase of absorption to reach or exceed the Lambertian limit. There are two typical ways to fabricate the increased scattering surface, including integrating a textured layer and preparing ordered structures. Such as the grating structure with an appropriate periodicity that meets the condition of Bragg scattering can make the light appear Bragg-scattered to enhance the light extraction (Bruetting et al., 2013; Cai and Qi, 2015).

## The Applications in Optoelectronic Devices

### Photodetectors

The photodetector is a kind of typical optoelectronic device, which can be adopted in many fields like clinical medical, aerospace industry, military project, communication, and sensors since photodetector can convert the light signal into electrical signals for followed processes. However, photodetector development still faces low conversion efficiency, while the micro/nanostructures can improve the light absorption by various strategies (Wang and Kim, 2017; Zhou and Huang, 2018). Among many structures, gratings play an essential role. For example, Hu et al. reported an excellent perovskite photodetector with nanograting through nanoimprint lithography (Figure 1C). The performance of photodetector was improved after grating imprinted. The width of fabricated nanograting was 270 nm, and the pitch was 600 nm, which could reduce reflectance during the entire spectrum. Meanwhile, the transmission also appeared to decrease in the range of 550–800 nm. In addition, perovskite film showed higher crystallinity under the appearance of nanograting. The combined effects lead to the improvement of ∼35 times in responsivity and ∼7 times in on/off current ratio (Wang et al., 2016). Besides the above traditional grating structure, more complex grating structures are applied in many devices gradually. Li et al. prepared a kind of moiré perovskite photodetector using a stacked dual shallow grating structure (Song et al., 2021). The dual grating structure induced feedback reflection, diffraction, and appearance of waveguide modes, resulting in the enhanced light-harvesting of the photodetector. Compared with flat perovskite photodetector, the detectivity and responsivity were improved by 7.8 and 6.7 times, respectively. In practical applications, the polarization states of light usually have more potential value. Hence, the detection of polarized light also is important. For example, Xia et al. reported a photodetector comprised of a stable 2D layered Ruddlesden–Popper perovskite arranged in grating structures. And the photodetector performed high responsivity of 3.5 AW^−1^, detectivity exceeding 1 × 10^15^ Jones, and a fast response with a rise time of 4.1 ms and a decay time of 3.3 ms. It is noticed that the photodetector could realize polarization detection, in which the photocurrent varies with polarization angle due to the different dielectric constants of the perovskite grating structures in different directions (Li et al., 2019).

### Solar Cells

The conversion of environmental energy into effective energy is more critical for modern society. The solar cell is an effective strategy in this field. Solar cells usually absorb photons to induce the generation and transport of charge carriers, and the electric energy was collected finally (Lewis, 2016; Polman et al., 2016). Although solar cells have been developed for many years, there are still many problems, including the path length of light absorption mismatched the diffusion length of photo-generated carriers. While the increase of path length of the light absorption layer means the thickness of the absorbing layer increased, limiting the portability and the cost. The ordered grating structures can solve the above problems by unique optical properties without changing the thickness of light absorbing layer. Mellor et al. reported an excellent crystalline silicon solar cell with diffraction gratings fabricated through nanoimprinted technology. As shown in Figure 1D, the grating structure enhanced the incident light absorption obviously, especially the crossed grating structure with the depth of 200 nm appeared the stronger ability in light absorption than linear grating with the depth of 300 nm. Firstly, weakly absorbed photons were trapped and deflected into oblique orders through the diffraction gratings on the rear-side. Secondly, the path of light absorption was extended efficiently because the incident light was trapped within the absorber layer by the total internal reflection of the front side (Mellor et al., 2013). As mentioned above, the perovskite with grating structures can improve the ability of light absorption and scattering. At the same time, the crystallinity of perovskite also was enhanced. Therefore, the patterned perovskite not only was applied in photodetector but also was applied in solar cells. Song et al. reported a new imprinting method to fabricate the solar cells using the perovskite with grating structures as the active layer. And the larger area grating structures were fabricated by commercial optical discs, which the size of grating depended on the area of the CD or DVD (The diameter of CD and DVD is 12 cm). The improved scattering and absorption abilities of solar cells can trap more incident light and suppress carrier recombination simultaneously. Utilizing CD or DVD as a mold would have different grating structures, for example, when CD as a mold leading to the grating period and line width was ∼1.5 and ∼1.0 µm, respectively. When DVD was a mold, the grating period and line width was ∼0.75 and ∼0.5 µm, respectively. And the heights of gratings were all ∼0.1 µm. Actually, the height of structures will influence the performance of devices, such as the continuity of perovskite film can be broken when the height was exceeded the threshold value. On the opposite, the light trapping effect will be limited by the low height. Therefore, it is essential to consider the range of height. Under the synergistic effect of the above results, the power conversion efficiency and photocurrent density of perovskite solar cells with grating structures compared with non-structures have been improved from 16.71 to 19.71% and 21.67 mA cm^−2^ to 23.11 mA cm^−2^. The authors have proved the grating structure has perfect homogeneity through the atomic force microscope images. Additionally, the stability of perovskite solar cells was also enhanced that the efficiency still keeps above 90% after one month exposure on air (Wang et al., 2018).

## Organic Light-Emitting Device

Organic light-emitting devices (OLEDs) are a kind of essential and representative optoelectronic devices, which have been applied in display and light panels with the advantages of color tunability, low cost, self-emitting property, and so on. However, there is an obvious problem in organic light-emitting devices, which is ∼80% generated photons are trapped in devices leading to low light extraction efficiency. It is noticed that the ordered micro/nanostructures within the OLEDs can induce the outcoupling effect of trapped photons and regulate the emitting properties to improve light extraction efficiency (Feng et al., 2017). During the internal light extraction processes, about 40% SPPs mode loss happened around the interface between organic layer and electrode. And the grating structure can relieve SPP loss. Therefore, the fabrication of micro/nanostructures on a metal electrode is important because the electrode has better stability. Ma et al. prepared grating structures on the ultrathin gold electrode through polymer-assisted thermal nanoimprint technology, and the fabricated gratings were 320 nm in period and 60 nm in depth. Taking the advantages of nanograting structures, the momenta of SPPs and photons could realize momentum compensation to improve light extraction and performance of OLEDs (Ma et al., 2020). For the white organic light-emitting devices (WOLEDs), broadband light extraction is deserved to devote the effort. As shown in Figure 1E, Sun et al. reported a kind of WOLEDs involved dual-periodic gratings, in which the maximum current efficiency was enhanced by 37% (from 16.27 cd/A up to 22.33 cd/A). In addition, compared to single periodic gratings, the dual-periodic gratings could broaden the SPP resonance (Bi et al., 2013). Besides the unpolarized light, the polarized light has become more and more important as an important and appealing functional expansion in practical applications. And the grating structures also was applied in OLEDs to emit linear polarized light. Zhou et al. prepared a series of aluminum and polyurethane acrylate nanograting structures on the green OLED substrate using developed soft nanoimprinting technology to emit linear polarized light. The devices produced an angle-invariant average extinction ratio as high as 20dB when the viewing angle within ± 60° due to both surface plasmons and cavity modes contributed to the TM-polarized light selection (Zhou et al., 2020). At the same time, the development of nanofabrication technology influences the light extraction efficiency of OLEDs to a considerable degree. At present, there are two typical methods to fabricate grating structures, including laser ablating method and nanoimprinting method. The laser ablation method takes advantages of simple processes and the adjustable period according to the applied laser wavelength to reach the smaller grating period. However, the substrate may be damaged by the high power of the laser during the ablation process. In contrast, the nanoimprint method can avoid the above damages because nanoimprint is a secondary transfer process. Therefore, compared to laser ablation, the nanoimprint method usually involves complex fabrication processes and the limitation of long periods. For example, Sun et al. utilized a simple one-step laser ablating method to prepare the OLEDs with periodic grating structures. The method of two interference beams was taken to avoid destroying the polymers because of the low ablation threshold. The grating structures play an important role in recovering power lost whatever in SPPs or waveguide mode, and the efficiency was proved enhanced three times finally (Bai et al., 2011).

### Lasers

For the rapid development of integrated photonic circuits or chips, a miniature laser source is necessary. Laser with perfect intensity and directionality can be emitted from lasers through stimulated emission of radiation and amplification. Organic-inorganic-perovskites can be applied in miniature lasers due to their excellent and unique properties, including tunable bandgaps. For example, Gu et al. firstly reported perovskite distributed feedback resonator with grating structures using thermal nanoimprint lithography, which proposed a new method for the design and fabrication of perovskite lasers. The resonator performed the ability of narrow amplified spontaneous emission (The full width half-maximum was 2.4 nm) even the pump power was only 0.1 W/cm^2^ and a 16-fold reduction than pristine thin film (Gharajeh et al., 2018). Actually, both light and electric sources can excite the lasers, such as Takenobu et al., who reported electroluminescence from a single-crystal light-emitting transistor (LET) with a grating resonator using the soft ultraviolet-nanoimprint lithography. And the electroluminescence could be controlled by the sub-micrometer grating structure. Moreover, the final realization of single-mode lasing depended on the Bragg diffraction and mode coupling distributed feedback (DFB) system. The above research overcame the combination problem between LET and DFB resonators (Maruyama et al., 2015). Except for frontier research, the lasers with grating structures have been applied in practical applications. Lin et al. reported a kind of hybrid plasmonic nanolaser for sensing applications, and the role of Al grating structures were plasmonic Bragg reflectors to decrease the mirror loss. As shown in Figure 1F, the nanolaser could serve as a refractive index sensor to detect glucose solutions. The sensitivity of the nanolaser was 249 nm/RIU under the resonant wavelength of 373 nm (Cheng et al., 2018). Strong light trapping ability will bring new paths for next-generation lasers.

## Conclusion and Outlook

In this minireview, we have summarized the mechanisms of grating structures in photon-related devices, including resonant effect, scattering enhancements, and plasmonic effect. Taking advantages of light management strategies of grating structures, the structures have been applied in many optoelectronic devices, such as photodetectors, solar cells, organic light-emitting devices, and lasers. Although the various devices have proved the important role of grating structures successfully. However, there are still many bottlenecks that need to be solved, including 1. how to improve the controllability of fabrication for grating structure; 2. how to balance the relationship between optical and electrical performance; 3. although the grating structure is more simple than others, but decrease the cost and simplify the preparing processes are still crucial for applications in business. Nevertheless, with the rapid developments of nanofabrication technologies, rational structure design, and advanced fundamental theories, more grating structures will be applied in excellent optoelectronic devices, which will bring our better daily life.
